# Road-based line distance surveys overestimate densities of olive baboons

**DOI:** 10.1371/journal.pone.0263314

**Published:** 2022-02-02

**Authors:** Christian Kiffner, Filipa M. D. Paciência, Grace Henrich, Rehema Kaitila, Idrissa S. Chuma, Pay Mbaryo, Sascha Knauf, John Kioko, Dietmar Zinner

**Affiliations:** 1 The School For Field Studies, Center For Wildlife Management Studies, Karatu, Tanzania; 2 Department of Human Behavior, Max Planck Institute for Evolutionary Anthropology, Ecology and Culture, Leipzig, Germany; 3 Junior Research Group Human‐Wildlife Conflict & Coexistence, Leibniz Centre for Agricultural Landscape Research (ZALF), Müncheberg, Germany; 4 Cognitive Ethology Laboratory, Germany Primate Center, Leibniz Institute for Primate Research, Göttingen, Germany; 5 Vassar College, Poughkeepsie, New York State, United States of America; 6 Tanzania National Parks, Conservation Science Unit (Veterinary), Arusha, Tanzania; 7 Institute of International Animal Health / One Health, Friedrich-Loeffler-Institut, Federal Research Institute for Animal Health, Greifswald, Insel Riems, Germany; 8 Infection Biology Unit, Germany Primate Center, Leibniz Institute for Primate Research, Göttingen, Germany; 9 Department of Primate Cognition, Georg-August-University of Göttingen, Göttingen, Germany; 10 Leibniz ScienceCampus Primate Cognition, Göttingen, Germany; U.S. Geological Survey, UNITED STATES

## Abstract

Estimating population density and population dynamics is essential for understanding primate ecology and relies on robust methods. While distance sampling theory provides a robust framework for estimating animal abundance, implementing a constrained, non-systematic transect design could bias density estimates. Here, we assessed potential bias associated with line distance sampling surveys along roads based on a case study with olive baboons (*Papio anubis*) in Lake Manyara National Park (Tanzania). This was achieved by comparing density estimates of olive baboons derived from road transect surveys with density estimates derived from estimating the maximum number of social groups (via sleeping site counts) and multiplying this metric with the estimated average size of social groups. From 2011 to 2019, we counted olive baboons along road transects, estimated survey-specific densities in a distance sampling framework, and assessed temporal population trends. Based on the fitted half-normal detection function, the mean density was 132.5 baboons km^-2^ (95% CI: 110.4–159.2), however, detection models did not fit well due to heaping of sightings on and near the transects. Density estimates were associated with relatively wide confidence intervals that were mostly caused by encounter rate variance. Based on a generalized additive model, baboon densities were greater during the rainy seasons compared to the dry seasons but did not show marked annual trends. Compared to estimates derived from the alternative method (sleeping site survey), distance sampling along road transects overestimated the abundance of baboons more than threefold. Possibly, this overestimation was caused by the preferred use of roads by baboons. While being a frequently used technique (due to its relative ease of implementation compared to spatially randomized survey techniques), inferring population density of baboons (and possibly other species) based on road transects should be treated with caution. Beyond these methodological concerns and considering only the most conservative estimates, baboon densities in LMNP are among the highest across their geographic distribution range.

## Introduction

Population size and population trajectories over time are key parameters for understanding the ecology of animal communities and guiding species-specific conservation efforts. In many habitats, particularly in tropical forests, primate species constitute a major component of animal communities and provide multiple ecosystem services as folivores, frugivores, seed dispersers, seed predators, pollinators, and as prey and predators [1, 2].

Primate taxa, such as baboons (*Papio* spp.), also play key ecological roles in savanna woodland habitats [3–6] and have numerous impacts on the ecosystem. The magnitude of these impacts is likely dependent on their absolute population size. Baboons can be highly effective seed dispersers, which becomes particularly obvious in the case of the invasive Neotropical prickly-pears (*Opuntia* sp.), which is spreading in many parts of Africa [7–9]. Moreover, the abundance of baboons and their ecological role might change in ecosystems where humans have drastically reduced their predators or competitors. For example, in three protected areas in Ghana, where apex predators such as lions (*Panthera leo*) and leopards (*P*. *pardus*) had been extirpated, the population density of olive baboons has increased manifold. Concomitantly baboons took over the role of mesopredators with negative impacts on smaller ungulates and other taxa [10].

Baboons are listed as Least Concern by the IUCN [11] and they are regionally central to human-wildlife conflicts [12, 13]. Baboons are also reservoir hosts for zoonotic disease agents (e.g., treponematoses [14] and tuberculosis [15]). Risk assessment, simulation, and management of disease outbreaks require realistic data on the absolute population size of reservoir species. Thus, monitoring baboon populations is key for understanding their population dynamics and associated ecological and epidemiological effects.

Lake Manyara National Park (LMNP), is a small protected area in northern Tanzania that is part of the greater Tarangire ecosystem. The park represents a site with available long-term data on the abundance of olive baboons (*Papio anubis*). Based on time series from 1959 to 2016, the density of olive baboons showed an apparent exponential increase between 2011 and 2016 (see Fig 2 in [16]). However, this manifold density increase (c. 14 fold greater density in the last decade compared to the first decade of the time series) coincided with a change in methodology from attempted total counts from 1959 to 2009 to line distance sampling along roads from 2011 to 2016. Due to this change in monitoring methods, it is unclear whether this apparent shift in population density has indeed occurred or is merely method-related. To better gauge the actual olive baboon density in LMNP, we here attempt to assess potential bias associated with line distance surveys carried out along roads and compare density estimates from this method with an alternative method to approximate the abundance of olive baboons; such comparative assessments are typically recommended when assessing potential biases of wildlife monitoring methods [17, 18].

Line distance sampling is frequently used for monitoring ungulates in East Africa [19, 20], and it is also a common tool for estimating the size of primate populations [21–27]. While this method explicitly accounts for imperfect detectability [28], line distance surveys are occasionally carried out along existing path or road networks due to safety concerns, restrictions on off-road driving in protected areas, or logistical or budgetary constraints associated with walking transects [29]. Extrapolating estimated densities from non-randomly distributed transects to the wider study area could, however, result in substantial sampling bias because roads generally do not represent the entire study area [30].

**Fig 1:**
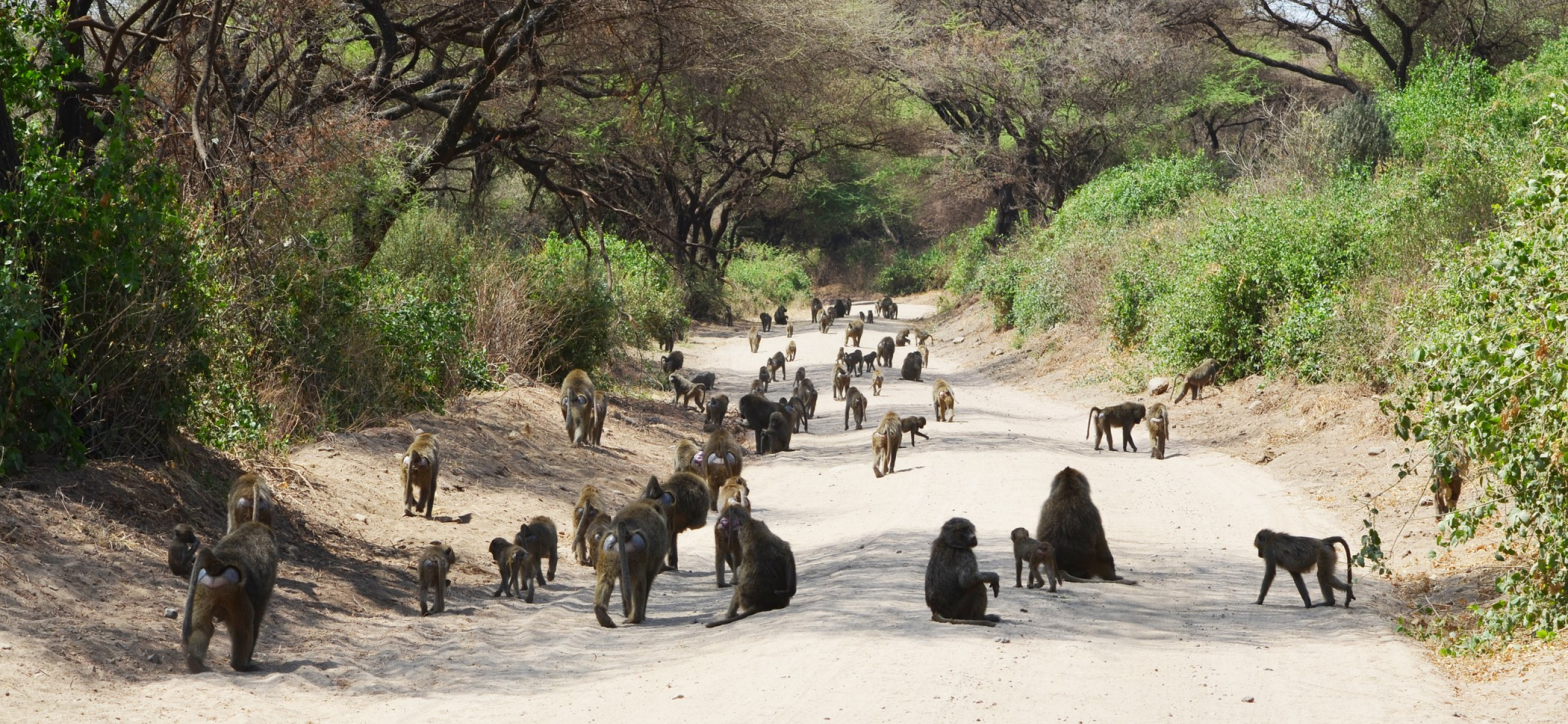
Part of an olive baboon (*Papio anubis*) group using a road in Lake Manyara National Park, Tanzania (Photo: Filipa M.D. Paciência).

Even though baboons are largely terrestrial and also use open habitats, counting baboons via total counts can be a veritable challenge. Due to limited visibility and constrained access, the key assumptions of total counts (i.e. all individuals are detected with certainty and no individuals are counted multiple times) are unlikely met. However, if it would be feasible to estimate the maximum number of social groups and the group size of olive baboons, it should be possible to establish an upper limit of olive baboon abundance in a given area. Olive baboons live in discrete, relatively large (up to 200 individuals) stable multi-male multi-female groups (Fig 1), and home ranges of neighboring groups overlap substantially [31–33]. Generally, baboon groups use tall trees or steep cliffs as sleeping sites to avoid predation [34]. Where cliffs are available, they are often preferred sleeping sites [35, 36]. Usually, a group uses more than one sleeping site within its home range, and the same site can be used several days in a row before switching sites [37–39]. These sites, in particular those in cliffs, are often used over multiple generations. The same sleeping site can be alternately used by more than one group; occasionally two groups use the same site simultaneously. Thus, even at high olive baboon densities, it is reasonable to assume that not all sleeping sites in an area are continuously occupied, i.e., there are usually more sleeping sites than social groups in an area. Therefore, the number of unique sleeping sites should represent the *maximum* number of social groups. Thus, while the method does not provide a precise estimate of the baboon density in a given area, multiplying the number of sleeping sites—which in the case of LMNP are mostly cliffs and well known by park rangers—with the average group size of baboons in the area should provide an upper limit for the abundance of baboons in LMNP.

In this paper, we first present density estimates of olive baboons based on line distance sampling carried out seasonally along roads from 2011–2019 and contrast these estimates with density estimates approximated by counting sleeping sites and the average size of social groups (the latter estimates should represent an upper limit for olive baboon density). Based on this comparison, we critically discuss monitoring methods for olive baboons and highlight possible implications of biased wildlife population assessments and high baboon densities in protected areas.

## Methods

Permission for conducting this research has been granted by the Tanzanian Wildlife Research Institute (TAWIRI) and the Tanzania Commission for Science and Technology (COSTECH; permits 2012-241-NA-2012-57 through 2020-130-NA-2013-191, as well as 2015-87-NA-2014-228, 2015-89-NA-2014-228, 2016-114-NA-2014-228 and 2016-115-NA-2014-228).

### Study site

**Fig 2:**
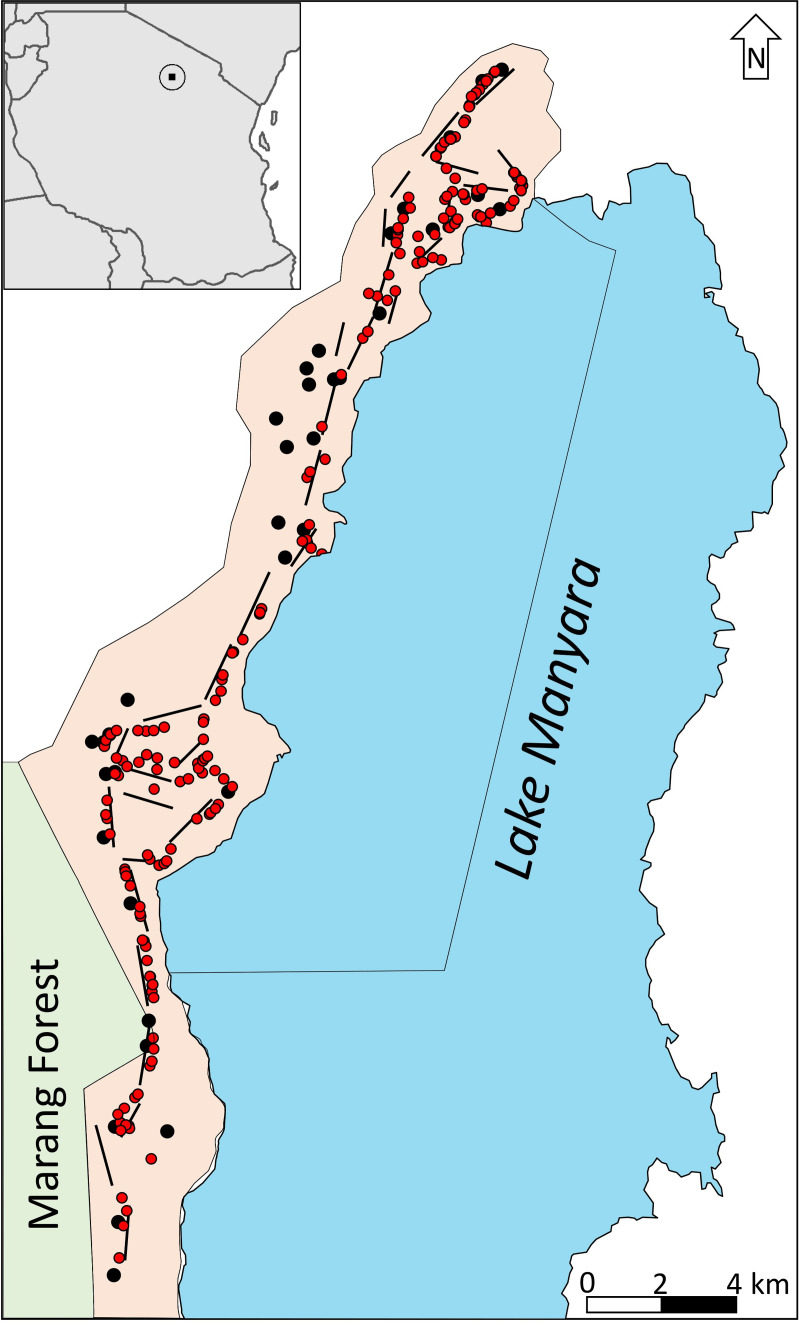
**Outline of Lake Manyara National Park (LMNP), positions of transects (straight lines), baboon sleeping sites (black dots), and baboon sightings during surveys from late 2015 to 2019 (red dots).** The boundaries of the park shown here depict the boundaries of the park before its extension in the southwest (Marang Forest) in 2012. The inset in the top-left indicates the location of the study area in northern Tanzania. All transects and sleeping sites are located inside the borders of LMNP.

The core area of LMNP (where we conducted our field surveys) is a narrow stretch of land (168 km^2^ in extent, centered approximately at 3.55°S and 35.78°E) located in the Eastern Rift Valley, in between the escarpment and the alkaline Lake Manyara (Fig 2). The climate is classified as semi‐arid to semi‐humid with a bimodal rainfall pattern characterized by the long rains from February to May, a dry season from June to October, and a short rainy season from November to December [40]; annual precipitation averages 600 mm and ranges from 430 to 1060 mm (data for 2011–2018 from the weather station at the LMNP headquarters). Relative fertile soils (mainly hardpan, plain soil, and clay) and a high groundwater table (facilitated by the water catchment of the Karatu highlands to the west of the park) ensure high vegetative productivity [41]. The vegetation is characterized by a patchwork of habitats including grasslands near the lake, Acacia (*Vachellia*) woodlands, and escarpment woodlands as well as lush groundwater forests and riverine vegetation [42, 43]. Since the late 1980s, the vegetation density in the bush layer of wooded habitats has increased substantially, and compared to historic baselines, densities of several large-bodied mammals have declined markedly over the last decades [16, 44].

### Line distance surveys

As part of a multi-species wildlife monitoring program in the ecosystem [45], we carried out road transect surveys in LMNP from 2011 to 2019. We used the existing road network because dense vegetation and off-road restrictions did not allow for a more systematic distribution of transects throughout the park. However, as the road network crosses the park from north to south, transects cover all major habitat types of LMNP. The roads are primarily used for tourism and the speed limit in the park is 50 km h^-1^. In general, motorists drive defensively and give wildlife the right of way.

Starting in the short rains 2011, we conducted three surveys per year to capture the main seasons; in 2019 we only conducted surveys during the long and the short rainy season. We conducted long rain surveys from the end of February until April; dry season counts between July and August and short rain counts between the end of September until the beginning of November. Short rains, therefore, mostly refer to the end of the dry season. Transects were 2 km in length and were separated by 500 m (measured by the odometer of the vehicle) to increase independence between transects. We drove along transects at slow speed (5 to 20 km h^-1^) with open-top vehicles and stopped the vehicle whenever trained observers (N = 3 to 9) detected olive baboons (or other target species). Upon detection, we identified the species, counted baboon individuals in a cluster, and measured the perpendicular distance between the transect and the approximate center of the cluster with a laser range finder (Bushnell Elite 1500).

To use a consistent definition for one animal cluster, we defined a cluster as individuals of the same species that were not separated by more than 50 m from other individuals of the same species; this distance is a common threshold for defining group sizes [46]. We differentiate between the size of a social group and the size of a cluster, whereby a cluster refers to the number of animals fulfilling the above definition and that can be a part of a social group. If olive baboons moved before the vehicle was in a perpendicular position relative to the transect and the animal cluster, we measured the distance to the initial location of the cluster [47]. With minor adjustments due to variable road conditions over time, we used the same transects during each of the 24 surveys (Fig 2). We carried out surveys between 7:30 am and 18:30 and completed each survey within one day using one to five vehicles. When using multiple vehicles simultaneously, each vehicle was assigned a particular, non-overlapping section of the park. Therefore, double counting due to baboon movement is unlikely to have biased density estimates. On a few occasions (n = 6), we carried out up to five surveys within a few days. In those cases, we combined the effort and baboon sightings of the same transect to estimate densities [47]; when repeating surveys within a few days, we varied the sequence of transects. Prior to all surveys, observers were trained in mammal species identification, line distance methodology, and field methodology, with particular emphasis on accurate distance measurements to the center of the animal and the initial location of the cluster and on exact counting of individuals in a cluster [28, 47–49].

We analyzed the data [50] in a distance sampling framework using the program DISTANCE 6.0 [28]. To obtain the required sample size (N = 60–80) for robust modeling of detection functions [47], we combined sightings across surveys and modeled global detection functions for the baboon sightings along road transects. We considered six candidate models to estimate the detectability of baboons: four conventional distance sampling (CDS) models where detectability is a function of perpendicular distance to the transect and two multiple covariate distance (MCDS) models. For the two MCDS models, we hypothesized that the detectability of baboons varies by season (i.e., lower visibility when vegetation is lush vs. dry; season defined as a categorical variable with three levels: dry season; short rains, long rains). For the CDS candidate models, we considered uniform, half-normal, negative exponential, and hazard rate key functions with cosine series expansion; for the MCDS candidate models we considered half-normal and hazard rate key functions with cosine expansion.

To select an appropriate detection model, we compared AIC scores of competing models and also considered the visual fit of the curve near the transect line and overall goodness of fit based on Kolmogorov-Smirnov test statistics [28]. In all models, we truncated 10% of the furthest detections, estimated cluster size based on the mean cluster size during each survey, and used the post-stratification setting to produce season-specific density estimates including associated 95% confidence intervals.

We used *R 3*.*6* [51] for additional data analysis and illustration of results. To assess broad temporal trends, we used the mean density estimates (derived from the chosen distance model) as a response variable in a generalized additive model (GAM), implemented in the *mgcv* package [52]. In this GAM, we modeled year as a smooth term (dimension of the basis *k* was set to 3 to allow sufficient wiggliness of the curve) and season as a categorical variable (three levels: dry season; short rains, long rains). To illustrate our results, we predicted the values of the GAM and overlaid the predictions with the observed density estimates using the *ggplot2* package [53].

### Approximating the abundance maximum of olive baboons and methods comparison

In 2015 and 2016, we systematically recorded the locations of all baboon sleeping sites in LMNP as part of ongoing field research on the behavioral ecology of *Treponema pallidum* subsp. *pertenue* (*TPE*) infected olive baboons [54, 55]. Sleeping sites were surveyed in the evening when groups were already near the sleeping areas and would not travel until the next day. Sites were located with the help of LMNP rangers, who have worked in the park for multiple years. Because sleeping sites of olive baboons are conspicuous and due to the extensive field experience of our field crew and the LMNP rangers, we are confident that we detected all sleeping sites of our study area.

When visibility was good, olive baboons in each social unit were counted by the same two observers. Otherwise, due to limited visibility and constrained accessibility, we assumed group sizes of olive baboons to range between 150 and 200 individuals, which might be at the upper end of group sizes in LMNP. This group size variation was based on total counts of the habituated study troop (170 individuals) and opportunistic observations of other baboon troops over a time period of 24 months in LMNP [54, 55]. We estimated the abundance of olive baboons by multiplying the value of the group size (lower bound 150; mean: 175, upper bound: 200) with the known number of sleeping sites (35, see Fig 2). Given that the number of sleeping sites might be slightly higher than the number of baboon groups and that the assumed group sizes are most likely also at the upper end, the estimated population size might be an overestimation of the “true” population size and thus represents an upper limit of the olive baboon abundance in LMNP.

We compared this abundance maximum with the mean abundance of olive baboons (± 95% confidence intervals) estimated by distance sampling along road transects from 2011 to 2019. Our population density estimate refers to the 168 km^2^ terrestrial area of LMNP and excludes the lake and the highland area of the Marang Forest [16].

## Results

### Line distance sampling

We detected clusters of olive baboons in each of the 24 seasonal surveys and detection functions were modeled based on 475 unique detections. Based on the Kolmogorov-Smirnov test, none of the tested detection function models fitted the observation data well (Table 1).

**Table 1:** Key parameters associated with six models to estimate detectability of olive baboons along road transects in Lake Manyara National Park (Tanzania).

Model	ΔAIC	P_a_	ESW (m)	KS p-value
CDS: hazard rate	0	0.048 (0.041–0.057)	4.8 (4.1–5.7)	≤0.001
CDS: negative exponential	167.4	0.089 (0.080–0.100)	8.9 (8.0–10.0)	≤0.001
CDS: half-normal	326.8	0.211 (0.194–0.231)	21.2 (19.5–23.1)	≤0.001
CDS: uniform	380.8	0.308 (0.289–0.328)	30.8 (28.9–32.8)	≤0.001
MCDS: hazard rate	387.2	0.322 (0.298–0.347)	32.2 (29.8–34.7)	≤0.001
MCDS: half-normal	462.2	0.370 (0.344–0.398)	37.0 (34.4–39.8)	≤0.001

“P_a_” is the estimated detection probability incl. associated 95%-confidence intervals, “ESW” is the estimated strip width in meters and its associated confidence intervals, and ‘KS p-value’ is the probability of a Kolmogorov-Smirnov goodness of fit test.

Model selection indicated that conventional distance models were more supported than MCDS models. The most supported detection function was the hazard rate key function, followed by negative exponential, half-normal, and uniform detection functions (Table 1).

**Fig 3:**
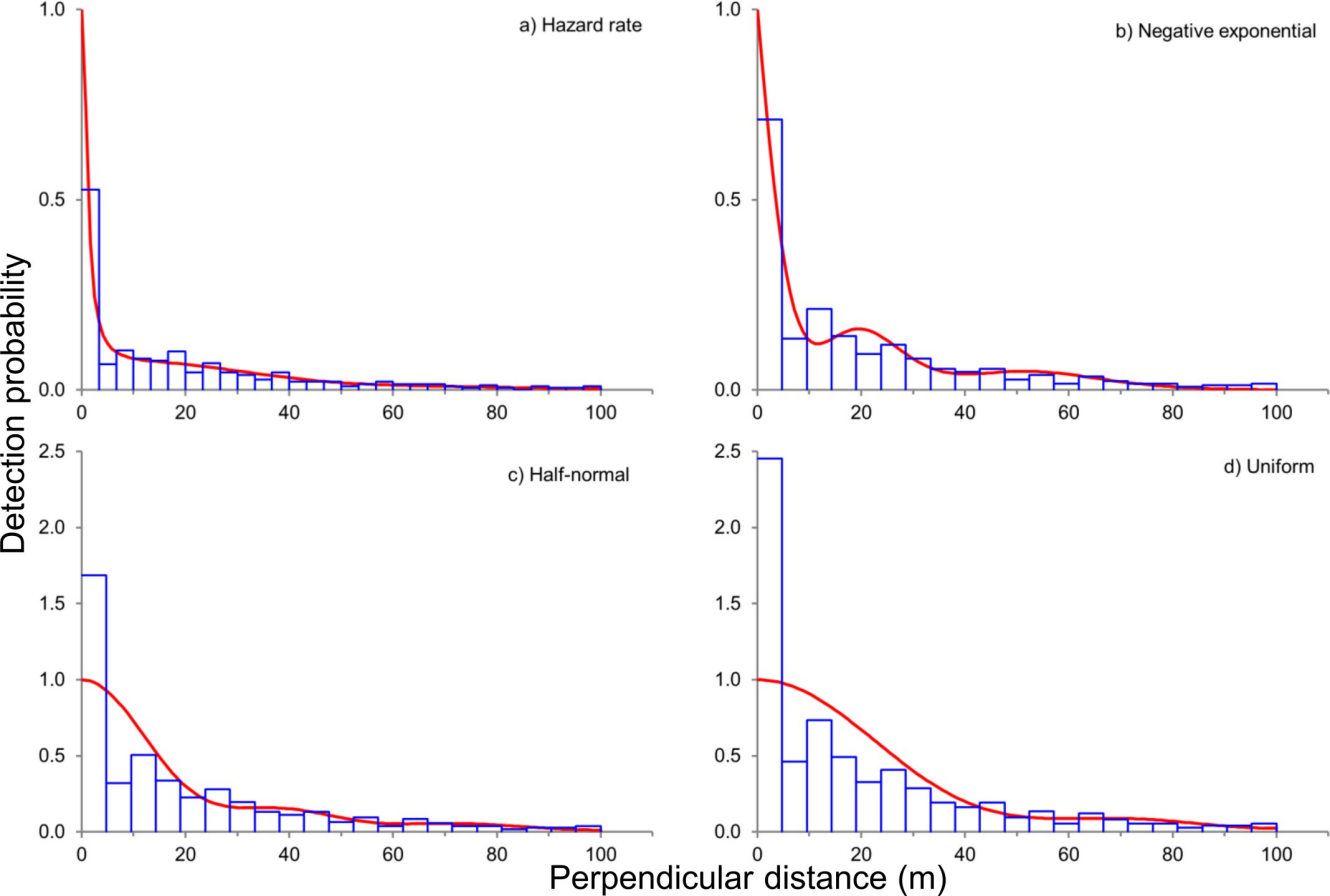
**Detection functions (red line) of conventional distance sampling models for olive baboons sighted along road transects in Lake Manyara National Park (Tanzania).** The histograms (blue bars) show the observed frequency of sightings in each distance bin; detection functions were modeled using a) hazard rate, b) negative exponential, c) half-normal, and d) uniform key functions with cosine series extensions.

However, hazard rate and negative exponential key functions–the two most supported models according to AIC scores–overestimated detectability near the transect line (Fig 3A and 3B; note the gap between fitted line and observed data), and estimated strip widths to be narrow (Table 1). Because these detection functions would result in very high (and possibly inflated) density estimates, we chose the half-normal detection model as a more conservative model to estimate seasonal density estimates (this model underestimated detectability near the transect line).

**Fig 4:**
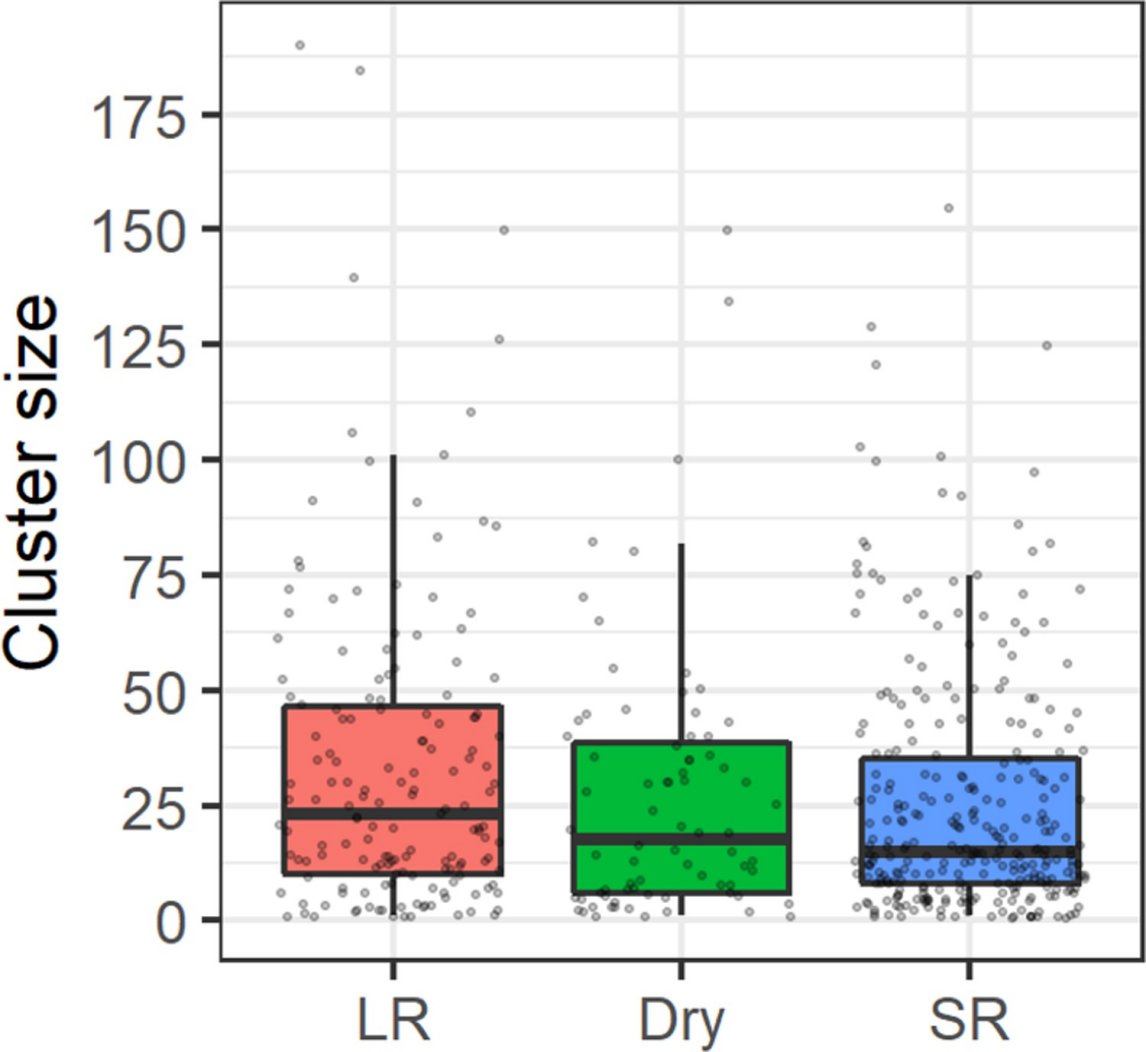
**Box-plots (bold midline indicates the median, the upper and lower limits of the box represent the third and first quartiles) of observed baboon cluster sizes during seasonal (LR: Long rains; Dry: Dry season; SR: Short rains) line distance surveys in Lake Manyara National Park (Tanzania).** The jittered grey points indicate observed cluster sizes.

The mean observed cluster size of baboon sightings was 28 (range = 1–190), and the observed cluster size did not vary significantly (p-values of negative binomial regression coefficients >0.092) across seasons (Fig 4); nevertheless, we used survey-specific cluster size estimates for extrapolating from cluster to animal density.

**Fig 5:**
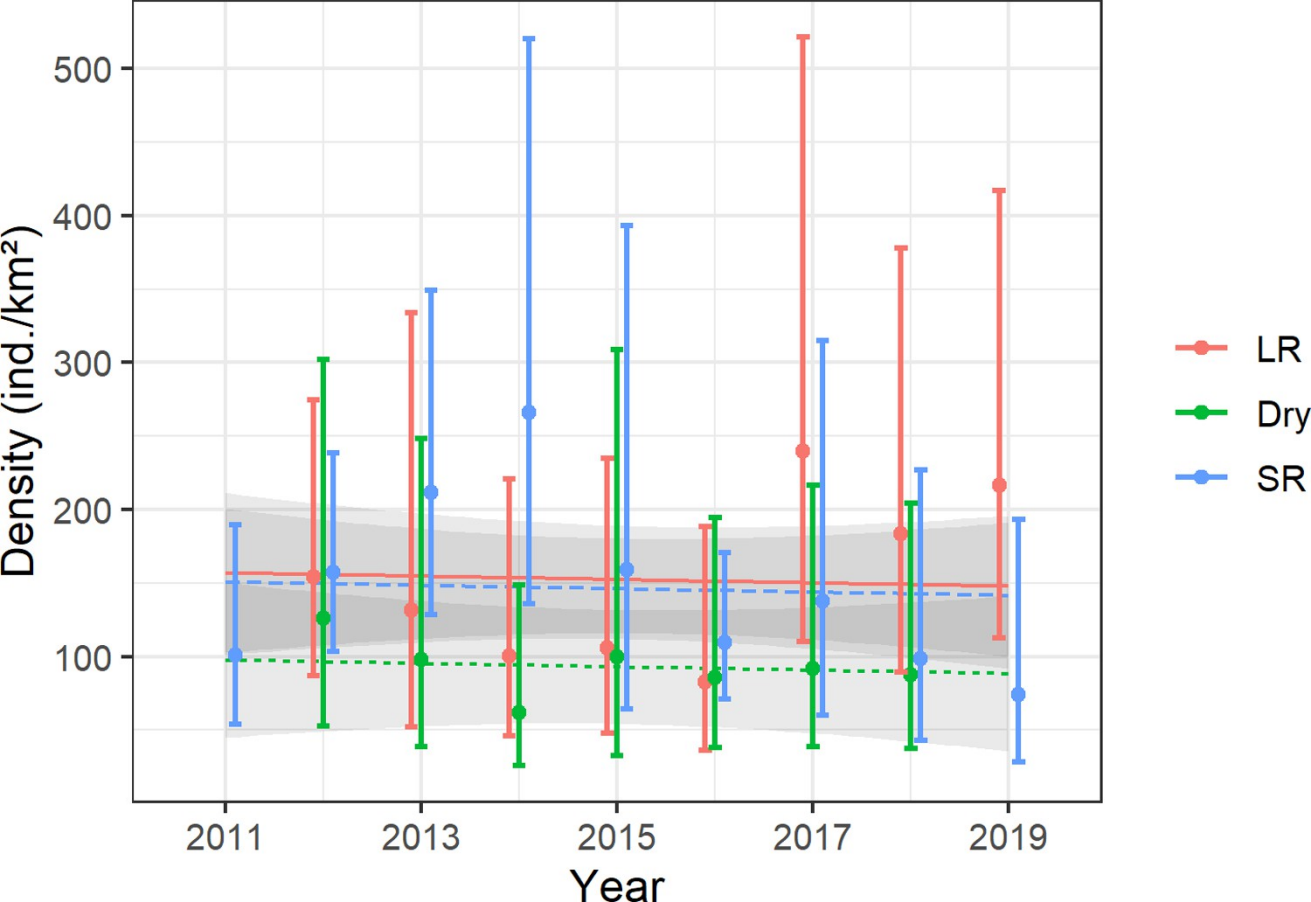
Seasonal (LR: Long rains; Dry: Dry season; SR: Short rains) density estimates and associated 95% confidence intervals of olive baboons in Lake Manyara National Park (Tanzania) from 2011–2019 (one survey in 2011; three surveys per year from 2012–2018, two surveys in 2019) and seasonal trend lines based on a general additive model.

Across the survey period, the mean baboon density was 132.5 individuals km^-2^ (95% CI: 110.4–156.2). Seasonal density estimates fluctuated considerably over time (Fig 5), yet we did not detect a marked yearly trend of baboon densities. Estimated baboon densities were substantially greater during the two rainy seasons than during the dry season counts, whereas this difference was significant for the long rains vs dry season comparison only (Fig 5; and see Table 2 for details of the GAM coefficients).

**Table 2:** Parameter estimates of a generalized additive model to describe annual and seasonal trends of olive baboon densities in Lake Manyara National Park, Tanzania.

**Parametric variable**	**Estimate**	**SE**	**t-value**	**p-value**
Intercept	152.237	18.561	8.202	≤0.001
Dry season (vs. long rains)	-59.458	27.175	-2.188	0.041
Short rains (vs. long rains)	-6.301	25.526	-0.247	0.808
**Smooth term**	**Estimated df**	**F-value**	**p-value**	
Year	1	0.063	0.805	

Confidence intervals associated with seasonal density estimates were relatively wide (Fig 5) and were primarily driven by variation in encounter rates (mean % contribution to estimated variation = 60%), and cluster size (mean % contribution to estimated variation = 39%). Uncertainty associated with the detection model (i.e., CDS detection model with half normal key function) accounted for one percent of the width of the estimated confidence intervals.

### Comparing olive baboon densities between survey methods

During fieldwork from 2015 to 2016, we recorded a total of 35 sleeping sites that were regularly used by olive baboon troops (Fig 2). Multiplied with the approximated mean troop size of 175 individuals (lower bound 150, upper bound 200), we estimated that the population maximum of olive baboons in LMNP was around 6,125 (range 5,250–7,000) individuals. This approximation represents the upper limit of olive baboon population size in LMNP, because not every sleeping site was occupied each night. Based on the distance sampling surveys, and extrapolating to the lowland surface area of LMNP (168 km^2^), the mean abundance of 22,260 baboons (95% CI: 18,547–26,746) was approximately 3.6 times greater than the sleeping site survey estimate.

## Discussion

By comparing two methods for estimating the density of olive baboons in LMNP, we show that method choice can result in manifold (~360%) differences in absolute density estimates of a common primate species. In the following, we will discuss the methodological aspects of our study and highlight the implications of our results.

### Monitoring primate populations

In line with Buckland *et al*. [47], we implemented a consistent field protocol for defining distinct cluster sightings (i.e., defined by a threshold distance of 50 m between individuals) and used laser range finders to estimate perpendicular distances between transect and the center of a baboon cluster. However, none of the tested detection functions were able to accurately describe the observed data near the transect line (see Fig 3 for poor fit of all four tested models at close perpendicular distances). This points to possible problems associated with the detection process and with the representativeness of the road transects for the wider study area. We assume that the observed spike in baboon detections on and near the transect is related to two, mutually non-exclusive reasons. First, when faced with (large) animal clusters near the transect, it may be difficult to accurately measure to the center of the cluster and it could be that observers then assigned those sightings as zero meters from the transect (in particular if baboons were detected on both sides of the road). Theoretically, measuring the perpendicular distance to each individual could overcome this issue [47], yet this may pose logistic challenges given the large group sizes and mobile nature of baboons. Beyond possible measurement issues, heaping of baboon sightings on or near the transect line (in our case, the road) could not be a measurement error but be caused by preferential use of roads by baboons. Indeed, high-resolution movement analyses of olive baboons in Laikipia (Kenya) suggest that baboons frequently followed roads and avoided dense vegetation [56]. As large parts of our study area were covered with very dense understory vegetation [16], the baboons’ propensity to use roads in LMNP may be even greater than in the relatively open savannah vegetation of Laikipia [56]. Thus, the high frequency of baboon sightings near the road transect may not necessarily be a measurement error but a consequence of the baboons’ spatial preference for moving along roads (Fig 1). While potential predators of olive baboons (e.g. leopards, lions, and spotted hyenas *Crocuta crocuta* [57–60]) may also frequently use unpaved roads in protected areas, these large carnivores are primarily nocturnal in LMNP [61]. As we carried out the line distance surveys between 8 am and 6 pm, olive baboons likely perceived moving along roads during these times not only as convenient but also as relatively safe.

As a consequence, such apparent local attraction to the road network may also partially explain the more than threefold discrepancy between road-based distance sampling estimates and the maximum olive baboon abundance based on the sleeping site count.

Beyond the relative local preference of roads, the use of roads as sampling transects could yield additional bias that operates at larger spatial scales. For example, if roads are not randomly distributed relative to habitat features, sleeping sites, and other areas that are preferred (or avoided) by olive baboons [56], extrapolating densities estimated along roads to the wider study area may further bias density estimates. Indeed, the largest component contributing to the confidence intervals of our density estimates was variation in encounter rates, contributing to 60% of the observed variance (range across surveys: 35 to 81%), suggesting that, during a given survey, baboon sightings occurred relatively unevenly across the surveyed area. While such spatial differences in local densities are to be expected in a group-living species at a given snapshot in time, mapping of olive baboon observations (Fig 2) from late 2015 to 2019, suggests that olive baboons principally used all (surveyed) parts of LMNP across the 2015–2019 period. In principle, if spatial information for all baboon sightings were available, one could model the seasonal density distribution of olive baboons as a function of environmental covariates and this could result in more accurate and more precise estimates [62]. Unfortunately, this was not possible because coordinates of baboon sightings were not recorded systematically during surveys prior to the short rain survey in 2015.

Overall, these considerations suggest that extrapolating olive baboon densities estimated along road-transects to the wider area overestimated olive baboon densities in our case study. Our findings further corroborate other comparative work on primate survey methods and highlight the magnitude of method-related differences for density estimates [e.g., [63, 64]]. In light of these potential sources of bias associated with density estimates derived from road counts, we assume that the density estimate of the sleeping site count (i.e., 31.3–41.7 baboons/km^2^) is a more realistic approximation for the absolute number of olive baboons in LMNP. While the sleeping site count method is also associated with substantial levels of uncertainty, this estimation method likely presents an upper limit for the baboon density in LMNP because the “true” number of social units may be somewhat lower than the number of sleeping sites. Camera trap distance sampling (implemented via a systematic sampling design) might help here to increase the accuracy of the estimates of baboon abundance. This method does not require the identification of individual baboons and proved to produce unbiased density estimates for other semi-arboreal and group living primate species [65].

Estimation bias associated with sampling along roads is not restricted to primates. For example, while sampling along roads yielded similar agouti (*Dasyprocta prymnolopha*) densities as sampling along systematically distributed transects, models fitted to road surveys differed in variance contribution, had lower detection probability and poorer fit [66]. For white-tailed deer (*Odocoileus virginianus*), distance sampling along roads yielded 3.0–7.6 times greater density estimates than systematic aerial surveys [17]. In the case of LMNP, contrasting road-based, distance sampling estimates with (likely more robust) estimates derived by either total count surveys (wildebeest *Connochates taurinus* and zebra *Equus quagga*) or mark-resight models (buffalo *Syncerus caffer*, elephant *Loxodonta africana*, and giraffe *Giraffa camelopardalis*) suggests that densities from road surveys can be biased low (e.g. for buffalo) or high (e.g. for wildebeest, zebra, giraffe, and elephant) [16]. Collectively, these comparisons suggest that naively extrapolating densities from road-based distance sampling to the wider study area can yield substantial bias.

While road-based surveys are potentially associated with biased absolute density estimates, temporal trends are unlikely to be affected by a road-based transect design, because we generally used the same transects across the study period and thus kept potential design-based bias constant. Thus, we are confident that the olive baboon population size in LMNP remained relatively constant from 2011 to 2019 (Fig 5).

#### Olive baboon densities in context

Even when considering the lowest approximation of the maximum olive baboon density (31.3 baboons km^-^^2^) or a sleeping site/social group ratio of 0.75 (this would yield an estimated range of 23.5–31.3 baboons km^-^^2^), baboon densities in LMNP appear high, particularly in the context of olive baboon densities in other parts of their geographic range (Table 3). However, comparing species-specific density estimates requires due caution because estimates have been derived with different methods and have been extrapolated to different spatial scales (i.e., home range scale vs. landscape scale).

**Table 3:** Population densities of *Papio anubis* across different sites in Africa. While providing a frame for comparison, please consider that density estimates are based on different methods, and often, the respective spatial scales to which the estimates refer (e.g., the home range of a primate group vs. landscape scale) are not always clear.

Site, Country	Ind./km^2^	Method [Reference]
Awash Valley, ETH	5.6	total count [67]
Bole Valley, ETH	26.0	total count [68]
Nairobi NP, KEN	3.9	total count [69]
Gilgil, KEN	10.3	total count [70]
QENP, UGA	17.3	total count [32]
Budongo Forest Reserve, UGA	11.0–14.0	line transect [71]
Kahuzi-Biega NP, COD	2.0	line transect [72]
Comoé, CIV	0.8–1.2	total count (roads) [73]
Pendjari NP, BEN	3.1 (95% CI: 1.4–6.6)	line transect [74]
Shai Hills, GHA	2.6–3.4	total count [4]
Arli NP, BFA	1.9	line transect (roads) [75]
Gombe, TZA	37.2	total count [33]
LMNP, TZA	132.5 (95% CI: 110.4–159.2)	this study, distance sampling
LMNP, TZA	36.5 (range: 31.3–41.7)	this study, sleeping site survey

BEN = Benin, BFA = Burkina Faso, CIV = Côte d’Ivoire, COD = Democratic Republic of the Congo, ETH = Ethiopia, GHA = Ghana, KEN = Kenya, TZA = Tanzania, UGA = Uganda, QENP = Queen Elizabeth National Park, LMNP = Lake Manyara National Park.

As indicated above, accurate information on baboon abundance is not only important in the ecological and conservation context, but also in the context of zoonoses. A higher population density is associated with increased intraspecific and interspecific contact rates and an increased level of competition for resources. This could favor the spread of communicable diseases and, if olive baboons move outside the boundaries of protected areas (e.g. in search for food resources), increase the risk for spillover transmissions to humans. Monitoring of these ecological processes is vital for risk assessment and pandemic preparedness. When census data of host species are combined with key epidemiological data (e.g. prevalence rates) the spread of infectious diseases such as the currently ongoing *TPE* epidemic [54, 76] within and across baboon groups in LMNP and other places in Sub-Saharan Africa can be simulated more accurately. Such studies are of fundamental importance for the identification of disease reservoirs [77] and in the case of LMNP, could provide further support for the elimination of yaws in nonhuman primates, the disease caused by *TPE* [78]. Finally, the census estimates from LMNP will help to understand the long-term consequences of treponematoses in its primate host. It is, for example, unclear whether LMNP’s olive baboon population is negatively affected by *T*. *pallidum* infections, which is a chronic disfiguring disease in humans and baboons [79].

### Hypothesized causes of high baboon densities

Unfortunately, uncertainty associated with both total counts conducted between 1959 to 2009 and with more recent olive baboon surveys prevents elucidating with certainty whether the olive baboon population has recently increased from 4.5 to 13.1 individuals km^-^^2^ (based on total counts [16, 44]) to around 30 individuals km^-^^2^ during the 2010s [this study]. Indeed, it is possible that these earlier “total counts” underestimated actual baboon abundance, as previous chief wardens of LMNP mentioned an “overpopulation” of olive baboons at least in the groundwater forest of LMNP during the late 1970s [80].

However, olive baboon density increases of several 100% within a few years are possible and have been observed in protected areas of Ghana. These increases have been linked to local extinctions of apex predators, consistent with predictions of the mesopredator release hypothesis [10]. As a recent camera trap study provides evidence that lion, leopard, and spotted hyena are relatively widely distributed across LMNP [81], it is unlikely that release from top-down control could have been the main driver of the apparent high population density of olive baboons in LMNP. Instead, we suggest that a combination of bottom-up (e.g., a favorable resource base) and top-down forces (e.g., persecution by humans outside the park) could explain the current (i.e. 2011–2019) high density of olive baboons in LMNP. Over the last decades, the boundaries of LMNP were gradually extended, adding more habitat and greater protection to baboons. For example, the southern extension to the park (annexed to the park in 1990) used to be a sugar cane plantation [82] and is now woodland habitat. At the same time, however, the human population grew substantially around LMNP, leading to an expansion of human settlements, agricultural areas, and substantial loss of natural habitat outside LMNP [83–85]. Given the vermin status of olive baboons in Tanzania, one may safely assume that in the past baboons were (and still are) persecuted by humans in communal lands. Collectively, this may have caused (1) an increase in baboon populations in areas where baboons were released from human persecution, and (2) a shift of olive baboon distribution away from human-dominated areas to the more secure national park [86]. Concurrent with shifts in mega-herbivore populations inside LMNP, the vegetation inside the park also changed considerably. In recent years, the understory of most bushland or woodland portions of LMNP is characterized by dense vegetation, which is in stark contrast to the previous predominantly open habitats of LMNP until the mid-1980s [16]. Likely, this bush encroachment creates very favorable conditions for olive baboons by providing sufficient food resources. In sum, these aspects provide a plausible, though not necessarily exhaustive, explanation for the observed high baboon densities in LMNP.

## Conclusions

This comparative baboon monitoring study provides evidence that road-based distance sampling overestimates densities of baboons, possibly because baboons are attracted to roads. Given the methodological challenges associated with monitoring primates (and other taxa) [48, 87], comparing monitoring methods with alternative estimation methods seems essential to assess, and ideally correct for potential biases. Beyond these methodological considerations, our monitoring suggests that LMNP is a stronghold for baboons.
